# Estimating heat-related mortality in near real time for national heatwave plans

**DOI:** 10.1088/1748-9326/ac4cf4

**Published:** 2022-02-03

**Authors:** Y T Eunice Lo, Dann M Mitchell, Ross Thompson, Emer O’Connell, Antonio Gasparrini

**Affiliations:** 1 School of Geographical Sciences, University of Bristol, Bristol, United Kingdom; 2 Cabot Institute for the Environment, University of Bristol, Bristol, United Kingdom; 3 Extreme Events and Health Protection Team, UK Health Security Agency, London, United Kingdom; 4 Department of Public Health Environments and Society, London School of Hygiene & Tropical Medicine, London, United Kingdom; 5 Centre for Statistical Methodology, London School of Hygiene & Tropical Medicine, London, United Kingdom; 6 Centre on Climate Change and Planetary Health, London School of Hygiene & Tropical Medicine, London, United Kingdom

**Keywords:** heatwaves, heat deaths, UK Health Security Agency

## Abstract

Heatwaves are a serious threat to human life. Public health agencies that are responsible for delivering heat-health action plans need to assess and reduce the mortality impacts of heat. Statistical models developed in epidemiology have previously been used to attribute past observed deaths to high temperatures and project future heat-related deaths. Here, we investigate the novel use of summer temperature-mortality associations established by these models for monitoring heat-related deaths in regions in England in near real time. For four summers in the period 2011–2020, we find that coupling these associations with observed daily mean temperatures results in England-wide heatwave mortality estimates that are consistent with the excess deaths estimated by UK Health Security Agency. However, our results for 2013, 2018 and 2020 highlight that the lagged effects of heat and characteristics of individual summers contribute to disagreement between the two methods. We suggest that our method can be used for heatwave mortality monitoring in England because it has the advantages of including lagged effects and controlling for other risk factors. It could also be employed by health agencies elsewhere for reliably estimating the health burden of heat in near real time and near-term forecasts.

## 1 Introduction

Heatwaves have caused severe impacts on human life around the world. More than 20 000 people died in the 2003 European heatwave [1], with 2234 excess deaths estimated to have occurred in England [2]. In 2010, the Indian city of Ahmedabad recorded an estimated 1344 excess deaths during a heatwave [3]. These devastating events, among many others, have led to the development of heat-health action plans at both national and sub-national levels in Europe [4], Ahmedabad [5], and other places.

The Heatwave Plan for England, which is delivered by UK Health Security Agency (UKHSA; formerly known as Public Health England) on behalf of agencies including the Met Office, the Department of Health and Social Care (DHSC), and the National Health Service (NHS), was introduced in 2004 [6]. The aim of this plan is to ‘prepare for, alert people to, and prevent, the major avoidable effects on health during periods of severe heat in England’ [6].

Underpinning the Heatwave Plan for England is the Heat-Health Alert System commissioned by UKHSA and operated by the Met Office. This system comprises five alert levels, ranging from Level 0 for year-round long-term planning, Level 1 for summer preparedness (in operation between 1 June and 15 September each year), to Levels 2–4 for various heatwave actions based on the probability of heatwave threshold conditions being met [6]. The NHS, health and social care providers, local authorities and the general public are all alerted within this system.

For example, when a Level 2 alert is issued, NHS England should get ready to hold health services to account for preparing for a heatwave, health and social care staff should check indoor temperatures regularly and prioritise high-risk people, and local authorities are advised to communicate the alert to staff, health and social care workers and the public. At Level 3, NHS England is advised to assemble mutual aid for local services, health and social care staff should take measures to reduce indoor temperatures and advise clients to access cool rooms, and local authorities are advised to mobilise community and voluntary support amongst other actions. A Level 4 alert has never been declared, but if the threshold conditions are met for it to be issued, the DHSC takes over responsibility for managing the heatwave at a national level, with multi-agency emergency response coordinated by central government. More details about the action plan can be found in the Heatwave Plan [6].

One of UKHSA’s responsibilities as set out in the Heatwave Plan is to monitor the impacts of high temperatures upon the population. UKHSA achieve this by utilising a range of syndromic surveillance systems to monitor near real-time impacts across the health service and estimate excess mortality. Since 2016, UKHSA have been publishing annual Heatwave Mortality Monitoring Reports on the number of allcause excess deaths in each of the nine regions in England during each heatwave every summer. These excess deaths are the difference between observed and expected deaths in the same summer [7]. Across all age groups and regions in England, ∼900–2600 heatwave excess deaths were reported in individual summers in the period 2017–2020 [2, 8]. These numbers reinforce why heatwave planning and mortality monitoring are important in the first place.

The association between high ambient temperatures and human mortality is well documented for global locations including the UK [9]. For regions in England, previous studies have found a non-linear increase in the risk of all-cause mortality above regional temperature thresholds, at a mean rate of 2.1% increase per ° C [10, 11]. However, this association varies between regions, with warmer regions showing a higher rate in increase in mortality above the regional threshold [10].

These regional temperature-mortality associations, usually established through quasi-Poisson regression coupled with a region-specific Distributed Lag Nonlinear Model (DLNM) that takes into account non-linear and lagged mortality responses to temperature exposures [12, 13], provide great information for assessing deaths attributable to heat. Previous studies have used DLNMs to attribute observed deaths to heat in the past [9, 10], estimate seasonal deaths [14], and project future heat-related deaths in various climate change scenarios [15, 16].

In this study, we investigate for the first time the use of regional temperature-mortality associations established with DLNMs in near real-time heat-related mortality monitoring. We estimate heat-related deaths during heatwaves for all nine England regions—North East England, North West England, Yorkshire and the Humber, the West Midlands, East Midlands, East of England, London, South East England and South West England—and compare them with the excess deaths estimated by UKHSA. This is the first study that evaluates both the epidemiological and UKHSA approach to heatwave deaths monitoring.

## 2 Methods

### 2.1 Data

We make use of daily all-cause death occurrences by regions in England for the period 2001–2018 obtained from the Office for National Statistics (ONS) [17, 18], as well as daily mean temperatures derived from the HadUK-Grid data set [19]. Specifically, regional daily mean temperatures are calculated by averaging daily minimum and maximum air temperatures obtained from the HadUK-Grid Climate Observations by Administrative Regions over the UK data set (v1.0.2.1) [20]. We use temperature data covering the period 2001–2020, with the 2020 data provided by the Met Office through personal communication, as they are not publicly available at the time of research.

For the purpose of mortality monitoring, heatwaves in England are defined by UKHSA Level 3 Heat Health Alerts and the mean central England temperature (CET) (see section 2.2). This definition is unique to UKHSA’s operational need for a pragmatic approach to define a heatwave period for analysis, and is different from the Met Office’s definition of a UK heatwave [21]. Throughout this study, we use the UKHSA definition because it has direct relevance to mortality monitoring within the Heatwave Plan for England.

Official UKHSA-defined heatwave periods for 2016–2020 are published in the annual UKHSA Heatwave Mortality Monitoring Reports [2, 8, 22–24]. To identify heatwave periods before UKHSA started annual reporting in 2016, we make use of a record of Level 3 Heat Health Alerts between 2011 and 2015, obtained from the UKHSA archive through personal communication, as well as the 2011–2015 daily mean CET series obtained from the Met Office HadCET data set [25, 26]. The CET is an instrumental record of temperature representative of a roughly triangular area enclosed by Lancashire, London and Bristol in the UK.

### 2.2 Definition of a heatwave

We follow the UKHSA definition and define heatwaves based on the following criteria:

(a) Days on which there was a UKHSA Level 3 Heat Health Alert, *or*
(b) Days with a mean CET greater than 20 ° C, and
(c) One day before and after the time period identified through the points above.

Only one of the first two criteria needs to be met to define a heatwave under this definition. Table A1 shows a breakdown of dates in the period 2011–2015 that meet each of these criteria. Table 1 lists all heatwaves identified for England for the period 2011–2020, including those published in the UKHSA reports (2016–2020) and those identified for the purpose of this study (2011–2015).

### 2.3 UKHSA’s method for estimating heatwave excess deaths

UKHSA estimate heatwave excess deaths by finding the cumulative difference between observed all-cause deaths and expected deaths over a heatwave period [7]. The observed deaths are corrected to account for delays between the date of death and the date of registration. For most years, the expected deaths are estimated by linearly regressing the observed deaths against time on all non-heatwave days during a chosen baseline period in summer of the same year [7]. These baseline periods are listed in table 1. For 2020, due to the outbreak of COVID-19, UKHSA estimated expected (baseline) deaths by averaging deaths from the seven non-heatwave days before and after each heatwave instead [2]. COVID-19 deaths, identified as either deaths with COVID-19 on death certificate or deaths for any reason within 60 days of a positive polymerase chain reaction (PCR) test, were excluded from the 2020 heatwave excess death estimates.

Since 2016 UKHSA have been publishing the number of excess deaths, along with its 95% confidence interval (CI), for both the under 65 and 65+ age groups, for each region in England during each heatwave. In the absence of ONS data on age-specific deaths by region (see section 2.1), we are unable to provide equivalent estimates using regression with DLNMs. Therefore, we sum the UKHSA excess death estimates across all age groups and use the resulting all-age totals throughout this study. Note that deaths from the under 65 age group make up a small proportion of total deaths. For example, 12% of the total deaths in 2020 came from the under 65 age group [2]. We find the CIs associated with the all-age totals by adding (or subtracting) the square root of the sum of squares of the upper (or lower) uncertainty in each age group to (or from) the central estimates. We note that this method is likely to underestimate the CIs of the all-age excess deaths, as it does not account for the uncertainty associated with the baseline above which excess deaths are calculated in each age group. A more appropriate method would be to calculate the standard error of the rate ratio. However, without the underlying observed and expected deaths data (we only have the excess values from the 2017–2020 UKHSA reports), we are unable to do this.

We extend the data set of regional all-age heatwave excess deaths in England to years before UKHSA started annual heatwave mortality reporting, i.e. 2011–2016, by following the UKHSA method and using daily all-cause death occurrences data obtained from the ONS. Since these are death occurrences rather than registrations, no delay correction is needed. Although UKHSA started reporting heatwave excess deaths by region and age group in 2016, excess deaths that are not statistically significant are not shown in that year’s report [22]. We calculate these numbers for 2016 by using the same method and baseline period mentioned in the report. For 2011–2015, we fix the baseline period against which excess deaths are calculated to 1 May to 30 September each year for simplicity. The 95% CIs are determined based on the standard deviation of deaths on non-heatwave days in the baseline period of the same year [7].

Throughout this study, we compare regional and England-wide all-age heatwave excess deaths estimated with this method to those estimated with DLNMs (see section 2.4). We find the England totals by finding the sum of all excess deaths in the nine regions in England. We find the CIs for these totals using the square root of the sum of squares of uncertainty method specified above. This summation approach is different from that adopted by UKHSA for mortality reporting in some years e.g. 2020, which involves running a separate regression model to estimate national expected deaths for excess deaths calculation [7].

### 2.4 DLNM-based method for estimating heat-related mortality

To assess mortality attributable to heat, or heat-related mortality, during individual heatwaves in each summer between 2011 and 2019, we make use of regional temperature-mortality associations established from the ten preceding summers and daily mean temperatures of that year. This means we apply regional associations found between daily mean temperatures and all-cause death occurrences in the summers of 2001–2010, to regional daily mean temperatures in the baseline period of 2011, in order to assess daily temperature-related mortality for 2011, for example. Here, we define summer as the period between 1 May and 30 September. For year 2020, we make use of temperature-mortality associations established from 2009 to 2018 and daily mean temperatures in 2020 due to a lack of 2019 ONS all-cause death occurrences data.

The temperature-mortality associations are established through quasi-Poisson regression with DLNMs, a technique that is well-used in the field of environmental epidemiology [12, 13]. These nonlinear associations are modelled by using a flexible natural cubic spline function with two internal knots at the 50th and 90th percentiles of the corresponding summer temperature distribution [27]. Flexible natural splines are chosen to allow for log-linear extrapolation to temperatures in any summer outside the range of those in the ten preceding summers [28]. A lagged response of up to 3 d after exposure is included by using unconstrained parameterisation in the lagresponse dimension of the DLNMs [27].

To control for residual seasonality and long-term trends, we include an indicator of day of week, a natural cubic spline function of day of year with four degrees of freedom, an indicator of year, and an interaction term between this spline and the year indicator in the regression model [27].

Cumulative temperature-mortality associations are then found across the whole lag period. We centre these associations at a region- and period-specific minimum mortality temperature (MMT), following the conventional approach in studies involving DLNMs [9, 28]. Since mortality risk is minimum at the MMT by definition, the MMT is considered to represent the optimum temperature of a place in a certain time period [9]. We refer to the MMT as ‘optimum temperature’ hereafter. For the UK, previous studies have found optimum temperatures at the 73rd [29] and 79th [30] percentiles of June to September temperatures. We, therefore, limit optimum temperatures to values above the 50th percentile of region- and periodspecific summer temperature distributions in this study.

Applying the regional, cumulative temperaturemortality associations to regional daily mean temperatures in each baseline period (see table 1) gives us time series of daily temperature-related mortality in that period. To further isolate the heat effect, we only count mortality on warm days, defined as days on which the mean temperature is above the corresponding optimum temperature [31]. We then sum all heat-related deaths in each region and heatwave for comparison with the UKHSA values.

We estimate uncertainty in heat-related mortality by using 100 additional temperature-mortality associations for each region and year of interest, following methods used in previous work [31]. These additional associations are generated by Monte Carlo simulations, under the assumption that the spline model coefficients follow a multivariate normal distribution. We report the 2.5–97.5th percentiles of heat-related mortality distribution as the 95% CI.

## 3 Analysis

**Figure 1:**
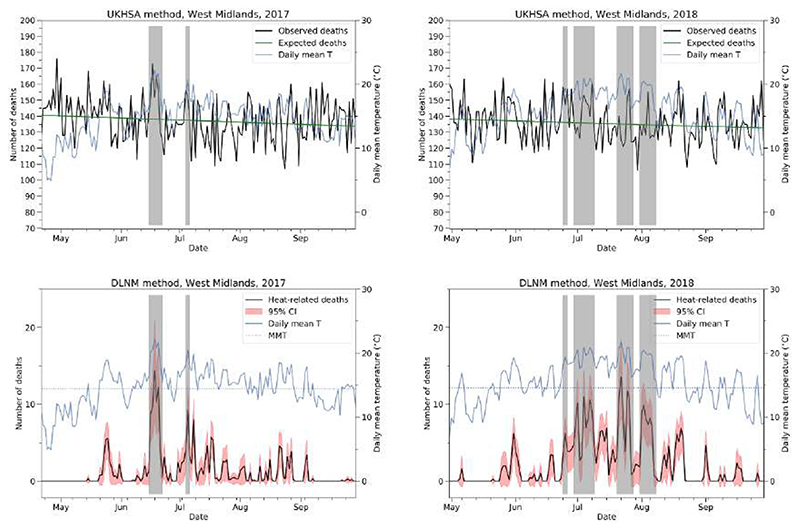
Top panel: time series plots of (black) observed all-cause death occurrences and (green) expected deaths in West Midlands over the baseline period in (left panel) 2017 and (right panel) 2018. Grey shading shows the official heatwaves (see table 1), and the blue lines show the regional daily mean temperature in 2017 and 2018, respectively. Bottom panel: time series plots of (black) daily heat-related deaths in West Midlands in the baseline periods in 2017 and 2018. Heat-related deaths are estimated from the respective temperature-mortality associations from summers in the previous decade and HadUK-Grid daily mean temperatures (blue solid lines) from the period of interest. Red shading shows the 95% CI of heat-related deaths. The blue dotted lines show the optimum temperature corresponding to the region and period, denoted here as MMT.

There were one to four heatwaves in England per summer in the period 2011–2020 (table 1). The top panel of figure 1 visualises how heatwave excess deaths are currently estimated by UKHSA, using examples from the West Midlands in 2017 and 2018. Expected deaths (green lines) are subtracted from observed all-cause deaths (black lines) and the resulting values are summed over individual heatwaves (grey shading) to give estimates of heatwave excess deaths.

**Figure 2:**
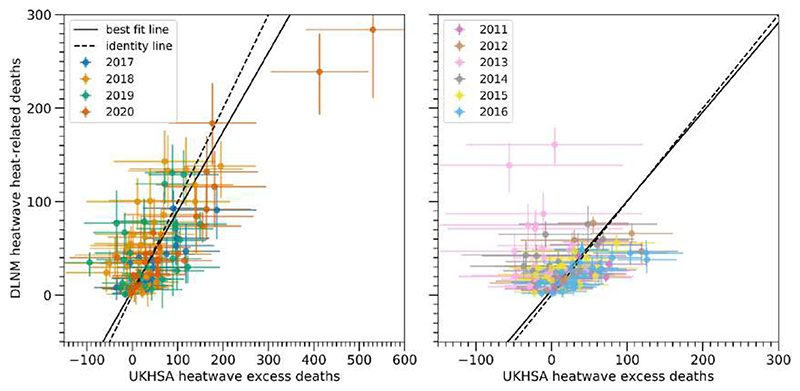
Heat-related deaths estimated from temperature-mortality associations versus heatwave excess deaths estimated by (left panel) UKHSA for 2017–2020 and (right panel) using the UKHSA method backdating to 2011–2016. Each dot represents one region in England during one heatwave (see tables 1 and A1). Error bars show the 95% CI. Best fit lines are shown by the black solid lines, whereas identity (1:1) lines are shown by black dashed lines.

In contrast, the method involving DLNMs estimates heat-related deaths, as illustrated in the bottom panel of figure 1. Figure 2 shows how these DLNM-based heat-related deaths compare with the UKHSA excess deaths in individual regions in England over individual heatwaves in the period 2011–2020. The two sets of numbers are consistent, with the best fit lines (black solid lines, found with linear orthogonal distance regression) closely aligned with the identity lines (black dashed lines). The slope of the best fit line for the period 2017–2020 is 0.85 (left panel), whereas that for the period 2011–2016 is 0.95 (right panel).

However, notable differences between estimates from the two methods are found, particularly for the years 2013 (right panel of figure 2) and 2020 (left panel). For year 2013, the two data points showing the largest deviations from the identity line correspond to London and South East England during the second heatwave. Our model estimates 161 (95% CI: 140–179) heat-related deaths in London, which is significantly higher than the corresponding backdated UKHSA estimate of 4 (95% CI: −113 to 121) excess deaths. For South East England, we estimate 139 (95% CI: 110–263) heat-related deaths, which is also significantly higher than the −56 (95% CI: −205 to 94) excess deaths estimated by UKHSA.

Time series analysis reveals a small peak in observed deaths relative to the level of expected deaths in London and South East England spanning the end of the first heatwave in 2013, between the first and second heatwaves (15 July; see table 1) and at the beginning of the second heatwave (see figure A1 for London; not shown for South East England). This is followed by a trough indicating deficit deaths (fewer observed deaths than expected deaths) in both regions in the second half of the second heatwave. This mismatch in timing between heatwaves and excess mortality, resulting in substantial inclusion of deficit deaths, explains the low UKHSA estimates for London and South East England in the second heatwave in 2013. These results suggest that the lagged mortality effect of heat, which is considered in our DLNMs but not in the UKHSA method, is important in impact monitoring.

**Figure 3:**
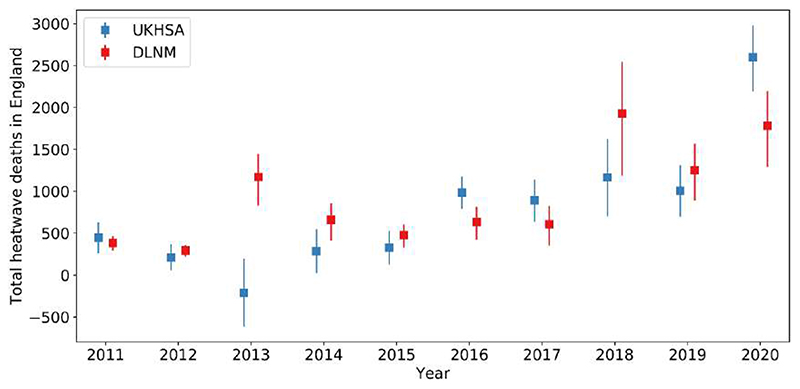
Total (blue) UKHSA excess deaths and (red) DLNM-based heat-related deaths across all regions in England and all heatwave periods in individual years between 2011 and 2020. Error bars show the 95% CIs.

The regional differences contribute to a significantly higher DLNM-based estimate of England total heatwave mortality, i.e. total heat-related mortality across all nine regions in England and all heatwaves in a year, compared to the equivalent UKHSA estimate for year 2013 (figure 3). Specifically, our DLNM-based method estimates 1171 (95% CI: 829–1443) total heat-related deaths, compared to the backdated UKHSA value of −211 (95% CI: −617 to 196). This result is consistent with the results from a previous study, which found nearly 1200 more heatwave deaths in England in 2013 using DLNMs based on 1993–2006 data than a UKHSA-style method [7].

Figure 3 shows that 2018 is another year where a substantial difference in England total heatwave deaths is found between the two methods. Our DLNM-based method estimates 1928 (95% CI: 1783–2050) total heat-related deaths, whereas UKHSA estimated 1165 (95% CI: 700–1623) excess deaths. This is due to more DLNM-based heat-related deaths than UKHSA excess deaths being estimated for most regions and heatwaves in 2018 (figure 2).

The summer of 2018 is the joint warmest in the UK’s observational record [32]. Not only was the summer season warm, it was also preceded by unusually high temperatures in April [33]. Since high temperatures are associated with high mortality risks, our DLNM-based method estimates the highest number of England heat-related deaths for 2018 amongst the studied years. However, UKHSA estimated similar numbers of excess deaths for 2016–2019. As seen in the example for London in figure A1, both the observed and expected deaths in 2018 are comparable to those in the other years. It is possible that the population was more cautious about high temperatures in the warmer- and longer-than-usual summer, leading to fewer deaths than would be expected from temperature alone. Future research is needed to unpick the reasons.

For summer 2020, we estimate statistically significantly fewer total heatwave deaths, at 1783 (95% CI: 1643–1908), compared to the UKHSA total of 2599 (95% CI: 2188–2981) excess deaths [2]. This difference is primarily driven by differences in mortality in South East England and London during the third heatwave in 2020. Our model estimates 284 (95% CI: 211–353) heat-related deaths in South East England, which is significantly lower than the corresponding UKHSA estimate of 530 (95% CI: 381–678) excess deaths (see left panel of figure 2). For London, we estimate 239 (95% CI: 193–280) heat-related deaths, which is also significantly lower than the 412 (95% CI: 305–520) excess deaths estimated by UKHSA.

Year 2020 is of particular interest to UKHSA because COVID-19 coincided with England’s highest estimated number of heatwave excess deaths since the introduction of the Heatwave Plan for England [2]. Since our DLNM-based results are solely based on 2009–2018 regional summer temperature-mortality associations and observed daily mean temperatures in summer 2020, all of which are independent of COVID-19, our significantly lower England total heatwave deaths estimate suggests that COVID-19 contributed to the high UKHSA heatwave deaths estimate.

One explanation of this discrepancy is that although UKHSA adapted their method of calculating excess deaths for year 2020 to separate the mortality effects of COVID-19 and heat (section 2.3), not all COVID-19 deaths were excluded in the calculations due to finite, albeit rapidly increasing, testing capacity that summer [34, 35]. However, it should be noted that COVID-19 death rate was substantially lower in June to August 2020 than the start of the pandemic (March 2020), even though testing capacity increased by two to four times in that period [34, 36].

Another plausible explanation is that COVID-19 acted as a risk amplifier for heat, either directly or indirectly [2]. This may be through increased exposure to indoor overheating and social isolation due to social restrictions, and reduced access or willingness to seek medical help during a public health crisis [37]. Future research is needed to examine the relative roles of under-reporting of COVID-19 deaths and amplification of heat-health risk by the pandemic in our 2020 results. One advantage of using DLNMs to estimate heat-related deaths during heatwaves, as is investigated in this study, is that risk factors including (but not limited to) COVID-19 can be controlled for in the main regression model. In fact, this has already been done in reverse in a recent study, which controlled for temperature-related mortality risk in the regression in order to estimate excess mortality due solely to COVID-19 [38]. By separating the heat impact from COVID-19, our method complements the existing UKHSA method in monitoring heatwave deaths, especially during a pandemic.

Among the studied years, our England total heatwave mortality estimate is within the 95% CI of the corresponding UKHSA estimate in 2011, 2012, 2015 and 2019 (figure 3). This means the two sets of England-wide estimates are consistent with each other in 40% of the studied years. While our method assumes that regional temperature-mortality associations from summers in the decade preceding a summer of interest is representative of that summer, the UKHSA method assumes linearity between expected deaths and time in individual summers [7]. Both methods are peer-reviewed, but they are based on different assumptions and statistical techniques.

## 4 Potential use in heat-health action plans

The existing UKHSA alerting and mortality reporting system was designed to focus on the acute response phase of episodes of heat from an operational stand point. By comparing observed deaths and expected deaths in the same summer, this method only requires a few months of daily death data and is, therefore, suitable for operational use and places that do not have a long record of daily death occurrences. A lot of these places are in low- and middle-income countries in Africa and South Asia that are most susceptible to heat [39].

However, for hundreds of global locations including regions in the UK, decades’ worth of daily mortality and temperature observations exist [16, 40]. Using England as an example, we have demonstrated that these data can be used to establish temperaturemortality associations specific to the places’ climates for heat-related mortality monitoring. This DLNM-based method has a few advantages over the UKHSA method. First, it includes lagged mortality responses to temperature exposures. Second, it controls for seasonality and trends in mortality, whereas the UKHSA method assumes linearity between time and mortality. Third, other time-varying risk factors such as COVID-19 [38] and air pollution [41] can be controlled for if needed. These advantages represent a substantial methodological improvement from the UKHSA method.

The studied framework is applicable to England as well as a wide range of other countries that have sufficient data. For England regions, future work should re-examine heat-related mortality during heatwaves in 2020 because the results shown here are based on 2009–2018, rather than 2010–2019, temperature-mortality associations. National agencies in and beyond England can also incorporate this framework into their heat-health action plans to examine heathealth impacts in different population groups, in order to allocate resources to reduce the impacts.

In addition to monitoring heat-related mortality in near real time, the temperature-mortality associations can be coupled with near-term weather forecasts to help alert organisations to the potential threat of any predicted heat events to public health. It is important that future work looks into heathealth forecasting and explores the possibility of further incorporating health into heat alert systems such as England’s Heat-Health Alert System, which is currently based on temperature thresholds that indirectly take into account the expected impact on mortality.

## 5 Conclusions

In conclusion, quasi-Poisson regression coupled with DLNMs can be used in a novel way to model regional summer heat-related deaths in near real time. Considering all nine England regions and heatwaves in 2011–2020 individually, this method provides heat-related mortality estimates that are broadly consistent with those estimated by UKHSA. However, discrepancies in some regions and heatwaves mean that the number of England total heatwave deaths estimated by using the DLNM-based method is only consistent with the corresponding UKHSA estimated range in four of the ten studied years. We suggest that this method can be used for national heatwave mortality monitoring in England, and that it is applicable to other countries’ heat-health plans where there are sufficient data.

## Supplementary Material

Appendix

## Figures and Tables

**Table 1:** Heatwave periods in 2011–2020 according to the UKHSA definition developed for mortality monitoring, along with the baseline periods against which excess heatwave deaths are calculated. Periods in and after 2016 are taken from the annual UKHSA Heatwave Mortality Monitoring Reports, whereas heatwave periods before 2016 are backdated using the same UKHSA definition (see section 2.2). Fixed baseline periods are used for years before 2016.

Year	Baseline period	Heatwave 1	Heatwave 2	Heatwave 3	Heatwave 4
2011	1 May–30 September	25 June–28 June	31 July–2 August	—	—
2012	1 May–30 September	17 August–20 August —	—	—
2013	1 May–30 September	11 July–14 July	16 July–24 July	31 July–2 August	—
2014	1 May–30 September	17 July–20 July	22 July–27 July	—	—
2015	1 May–30 September	29 June–2 July	21 August–23 August	—	—
2016	24 May–30 September	19 July–21 July	23 August–25 August	14–16 September	—
2017	22 April–30 September	16 June–23 June	5 July–7 July	—	—
2018	1 May–30 September	25 June–27 June	30 June–10 July	21 July–29 July	1 August–9 August
2019	3 May–12 September	28 June–30 June	21 July–28 July	23 August–29 August	—
2020	7 d before & after	23 June–27 June	30 July–1 August	5 August–15 August	—

## Data Availability

Daily all-cause death occurrences by regions in England are available from the Office for National Statistics, at www.ons.gov.uk/peoplepopulationandcommunity/birthsdeathsandmarriages/deaths/adhocs/005459dailydeathoccurrencesenglandregionsofenglandandwales1970to2014 and www.ons.gov.uk/peoplepopulationandcommunity/birthsdeathsandmarriages/deaths/adhocs/11189dailydeathsenglishregionsandwales2015to2018occurrences. The HadUK-Grid Climate Observations by Administrative Regions over the UK data set v1.0.2.1 is available at https://catalogue.ceda.ac.uk/uuid/e091188f36ff41fcae8c30da1ae77ea0. The HadCET data set is available at www.metoffice.gov.uk/hadobs/hadcet/index.html.
