# Supplementary material for: Estimating heat-related mortality in near real time for national heatwave plans
Source: Environ Res Lett. Author manuscript; Available in PMC 2022 Mar 24. (PMC7612535; doi:10.1088/1748-9326/ac4cf4)
Supplement: Appendix [file EMS143872-supplement-Appendix.pdf]

# Appendix

**Table A1.** Dates on which a UKHSA Level 3 heat health alert was issued or the mean CET was greater than 20 °C in the period 2011–2015. The rightmost column shows the heatwave periods identified based on these criteria and one day before and after.

| Year | Level 3 heat health alert | Mean CET > 20 °C | Heatwave period |
|---|---|---|---|
| 2011 | — | 26 June–27 June | 25 June–28 June |
| | — | 1 August | 31 July–2 August |
| 2012 | 19 August | 18 August | 17 August–20 August |
| 2013 | 12 July–13 July | 13 July | 11 July–14 July |
| | 17 July–19 July, 22 July–23 July | 17 July–19 July, 22 July–23 July | 16 July–24 July |
| | — | 1 August | 31 July–2 August |
| 2014 | — | 18 July–19 July | 17 July–20 July |
| | — | 23 July–26 July | 22 July–27 July |
| 2015 | 1 July | 30 June–1 July | 29 June–2 July |
| | — | 22 August | 21 August–23 August |

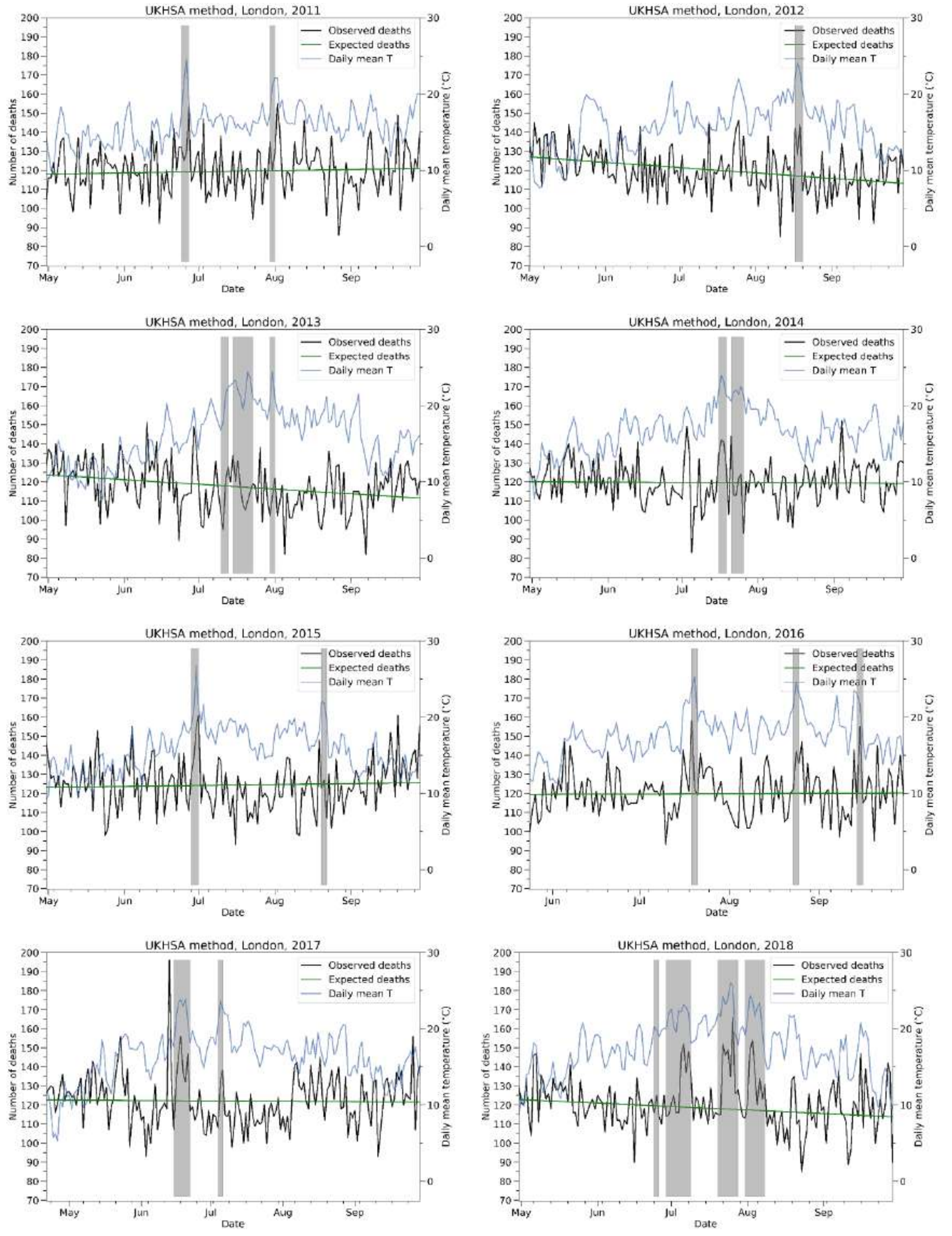


**Figure A1.** Time series plots of (black) observed all-cause death occurrences and (green) expected deaths in London over the baseline periods in 2011–2018. Grey shading shows the official heatwaves, whereas the blue lines show the regional daily mean temperatures.
